# Leg Ulcers Associated with Anagrelide

**DOI:** 10.4274/tjh.galenos.2021.2021.0399

**Published:** 2021-12-07

**Authors:** Tuba Oskay, Mehmet Özen

**Affiliations:** 1 Bayındır Health Group, Department of Dermatology, Ankara, Turkey; 2 Bayındır Health Group, Department of Hematology, Ankara, Turkey

**Keywords:** Anagrelide, Leg ulcers, Essential thrombocytemia

## To the Editor,

**Figure 1:**
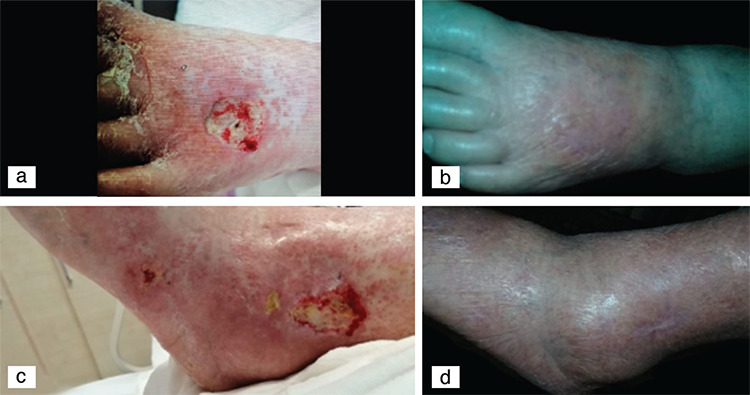
a) Ulcer on the back of the left foot, b) Healed lesions on the left foot, c) Ulcers on the lateral malleolus and lateral side of the left foot, d) Healed ulcers on the left foot.

A 77-year-old woman was referred to the dermatology department with a complaint of leg ulcers that had existed for a year. She had been treated with oral anagrelide at 0.5 mg twice a day for two years with the diagnosis of essential thrombocytosis (ET). Besides itching in her hands and legs, she had advanced non-healing leg ulcers that made walking difficult. A dermatologic examination revealed ulcers of 2x2 cm with demarcated, regular margins on the back of the left foot and ulcers of 2x1.5 cm covered by sloughing necrotic tissue over the lateral malleolus of the left foot, as well as punched-out tiny ulcers on the lateral sides of both feet (Figures 1a and 1b). She also had comorbidities of chronic obstructive lung disease, hypertension, and deep vein thrombosis.

Her physical examination was unremarkable except for mild splenomegaly. Laboratory investigations including vasculitis and metabolic work-up showed no abnormalities. The complete blood count test revealed hemoglobin of 12 g/dL, a white blood cell (WBC) count of 7710 µL, and a platelet count of 285000/µL (pretreatment platelet level: 685000/µL). The WBC differential counts were 77.2% neutrophils, 12.1% lymphocytes, 7.1% monocytes, and 2% eosinophils. Serum lactate dehydrogenase, C-reactive protein, and erythrocyte sedimentation rate were 265 (normal range: 60-200 U/L), 12 (normal: 6 mg /L), and 18 mm/h (normal: 20 mm/h), respectively.

Bacterial and fungal cultures showed no growth. Doppler ultrasound examination of the arterial and venous systems was normal. The ulcers were resistant to surgical debridement, various topical wound dressings, and antibiotic treatment. As the leg ulcers were thought to be caused by anagrelide use, the causative drug was discontinued and aspirin was given to control the platelet level. After three months, her lesions had significantly improved (Figures 1c and 1d). During two years of follow-up, no recurrence was noted.

ET is a myeloproliferative disorder characterized by prolonged peripheral thrombocytosis as well as thrombosis and hemorrhage susceptibility. Despite the fact that ET is characterized by skin involvement, therapies for ET also result in cutaneous manifestations. Although they are generally well tolerated, long-term treatments frequently cause cutaneous and mucosal side effects [1,2].

Anagrelide has been preferred as a platelet-lowering drug in patients with myeloproliferative neoplasms in recent years due to its rapid onset of action and selective thrombocytopenic effect. It works by preventing the maturation of platelets from megakaryocytes, reducing platelet production, having a selective effect on megakaryocytes, and preserving myeloid and erythroid lineages. Although anagrelide is known to be a phosphodiesterase (PDE) inhibitor that prevents platelet aggregation, its exact mechanism of action is unclear [3,4].

Clinical trials have shown that anagrelide is just as effective as other medications in reducing platelet counts without additional complications [5]. Anagrelide appears to have a higher response rate and may be better tolerated than other therapies. Furthermore, anagrelide is not mutagenic and there has been no evidence of leukemogenicity when compared to other drugs [3,4,5].

The most frequent side effects of anagrelide are headaches and tachycardia, which are caused by its inhibitory actions on PDE 3. The majority of frequent side effects are dose-dependent, low to moderate, and easily tolerable. Anagrelide-related cutaneous manifestations such as a transient rash, hair loss, skin discoloration, itching, and xerosis have rarely been reported. To our knowledge, only two previous cases of leg ulcers in anagrelide-treated patients have been published [6,7]. However, because one of those patients had previously taken hydroxyurea (HU), it is unclear whether anagrelide caused the leg ulcer. HU-related painful leg ulcers have been documented frequently [2,8,9].

Although the underlying cause of these leg ulcers remains unknown, they may be due to microcirculation difficulties and the effects of PDE inhibitors on skin vascularity. The presence of a temporary relationship and the elimination of other possible causes support the diagnosis of drug-induced leg ulcers with treatment focused on discontinuing the drug, as in our case. We believe that early diagnosis of anagrelide-induced leg ulcers is essential for these individuals to avoid functional and psychological problems.
